# An Atypical Case of CREST Syndrome With Early Complete Clinical Manifestation

**DOI:** 10.7759/cureus.86500

**Published:** 2025-06-21

**Authors:** Geil A Schock, Dana Simon, Ethan Weitzman, Raymond Weitzman

**Affiliations:** 1 College of Osteopathic Medicine, Michigan State University, East Lansing, USA; 2 William Beaumont School of Medicine, Oakland University, Auburn Hills, USA; 3 Rheumatology, Gary Burnstein Community Health Clinic, Pontiac, USA

**Keywords:** auto immune disease, crest syndrome, cutaneous calcinosis, internal medicine and rheumatology, limited cutaneous systemic sclerosis, limited scleroderma, sclerodactyly, social determinants of health (sdoh), systemic sclerosis (ssc), telangiectasia

## Abstract

CREST syndrome, the limited cutaneous subtype of systemic sclerosis, is defined by five classic clinical features: calcinosis, Raynaud’s phenomenon, esophageal dysmotility, sclerodactyly, and telangiectasia, which also forms the initialism. While diagnosis requires the presence of only three criteria, the full expression of all five is uncommon and typically develops gradually over many years. We describe the case of a 44-year-old Spanish-speaking female patient who presented to a community-based health clinic. Her initial evaluation identified the recent onset of Raynaud’s phenomenon, esophageal symptoms, sclerodactyly, and facial telangiectasia. Serology was notable for positive anti-centromere and antinuclear antibodies. One year later, she developed a painful mass on her right foot, ultimately identified as dystrophic calcification consistent with calcinosis. This case highlights a rare, rapidly progressive, and complete manifestation of CREST syndrome. This atypical presentation also underscores the importance of early recognition, multidisciplinary management, and careful attention to social determinants of health in patients with autoimmune disease.

## Introduction

CREST syndrome, or limited cutaneous systemic sclerosis (lcSSc), is a distinct clinical subtype of the autoimmune disease systemic sclerosis (SSc) [1,2]. CREST is an initialism based on the following diagnostic criteria, the presence of three of which is required for diagnosis: calcinosis, Raynaud’s phenomenon, esophageal dysmotility, sclerodactyly, and telangiectasia [1,2]. As opposed to the more severe diffuse cutaneous systemic sclerosis (dcSSc), CREST syndrome is associated with more calcium deposition in the skin (calcinosis), a higher risk of pulmonary fibrosis and pulmonary hypertension, but less widespread skin involvement. The pathophysiology of SSc is complex and characterized by an initial vascular insult that triggers platelet activation along with thrombotic and fibrinolytic cascades, ultimately leading to the development of autoimmunity and tissue fibrosis [1]. 

Further differentiating it from dcSSc, CREST syndrome is characteristically associated with anti-centromere antibodies as opposed to anti-Scl-70 and anti-RNA polymerase III [2,3]. Around 90% of SSc patients also present with a positive antinuclear antibody (ANA) [1,2]. SSc predominantly affects females, with a reported female-to-male ratio of approximately 5:1 and an earlier disease onset in women [1]. Global prevalence ranges from 38 to 341 cases per million persons, with annual incidence rates between 8 and 56 new cases per million [1]. The symptomatic progression of CREST syndrome typically occurs over many years, with Raynaud’s phenomenon often preceding other manifestations [1,4]. Studies have reported a mean delay of over five years between the onset of Raynaud’s phenomenon and the first non-Raynaud’s symptom in female patients with CREST syndrome [5]. Here, we describe an unusual presentation in which the full spectrum of diagnostic criteria emerged within one year of initial evaluation.

## Case presentation

Initial evaluation

A 44-year-old Spanish-speaking female patient presented to a community-based clinic for medically underserved populations with the recent onset of symptoms consistent with mild, episodic Raynaud’s phenomenon, followed by gastroesophageal reflux and dysphagia. Her past medical history included hypothyroidism, type 2 diabetes mellitus, diverticulosis, and recently treated endometrial carcinoma.

On physical examination, telangiectasia was present on her face, and sclerodactyly was noted involving the proximal upper and lower extremities. Autoimmune etiology was suspected due to her widespread and specific constellation of symptoms, with SSc leading the differential.

Laboratory testing revealed high-titer positive ANA (>1:1280) and anti-centromere antibody levels. Further autoantibody testing was negative for anti-Scl-70, anti-RNA polymerase III, dsDNA, anti-Smith, rheumatoid factor, and ribonucleoprotein (RNP) antibodies (Table 1). Values for other tests, such as C-reactive protein (CRP), erythrocyte sedimentation rate (ESR), C3 and C4 complement, total creatine kinase, and thyroid-stimulating hormone (TSH), all fell within normal limits. Due to the concern over possible interstitial lung disease and pulmonary hypertension with a likely diagnosis of CREST syndrome, an early high-resolution chest CT (HRCT) was conducted with normal findings. 

**Table 1:** Autoimmune panel results (units and reference ranges as reported by laboratory)

Autoantibody	Patient Value	Reference Range
Antinuclear (ANA)	Positive (>1:1280)	Negative (<1:80)
Anti-centromere	Positive	Negative
Anti-Scl-70	Negative (<0.6)	<7.0 U/mL
Anti-RNA polymerase III	Negative (<20)	<20 Units
dsDNA	Negative (<0.6)	<10 IU/mL
Anti-Smith	Negative (<0.7)	<7.0 U/mL
RNP	Negative (<2.5)	<5.0 U/mL
Rheumatoid factor (RF)	Negative (<15)	<15 IU/mL

Based on the patient’s clinical presentation and indicative serology results, a diagnosis of CREST syndrome was established. Given her mild symptoms and financial insecurity, conservative symptom management with over-the-counter antacid therapy for gastroesophageal reflux was recommended. She received extensive counseling on the importance of regular follow-up and pulmonary screening.

Emergence of final diagnostic criterion

**Figure 1:**
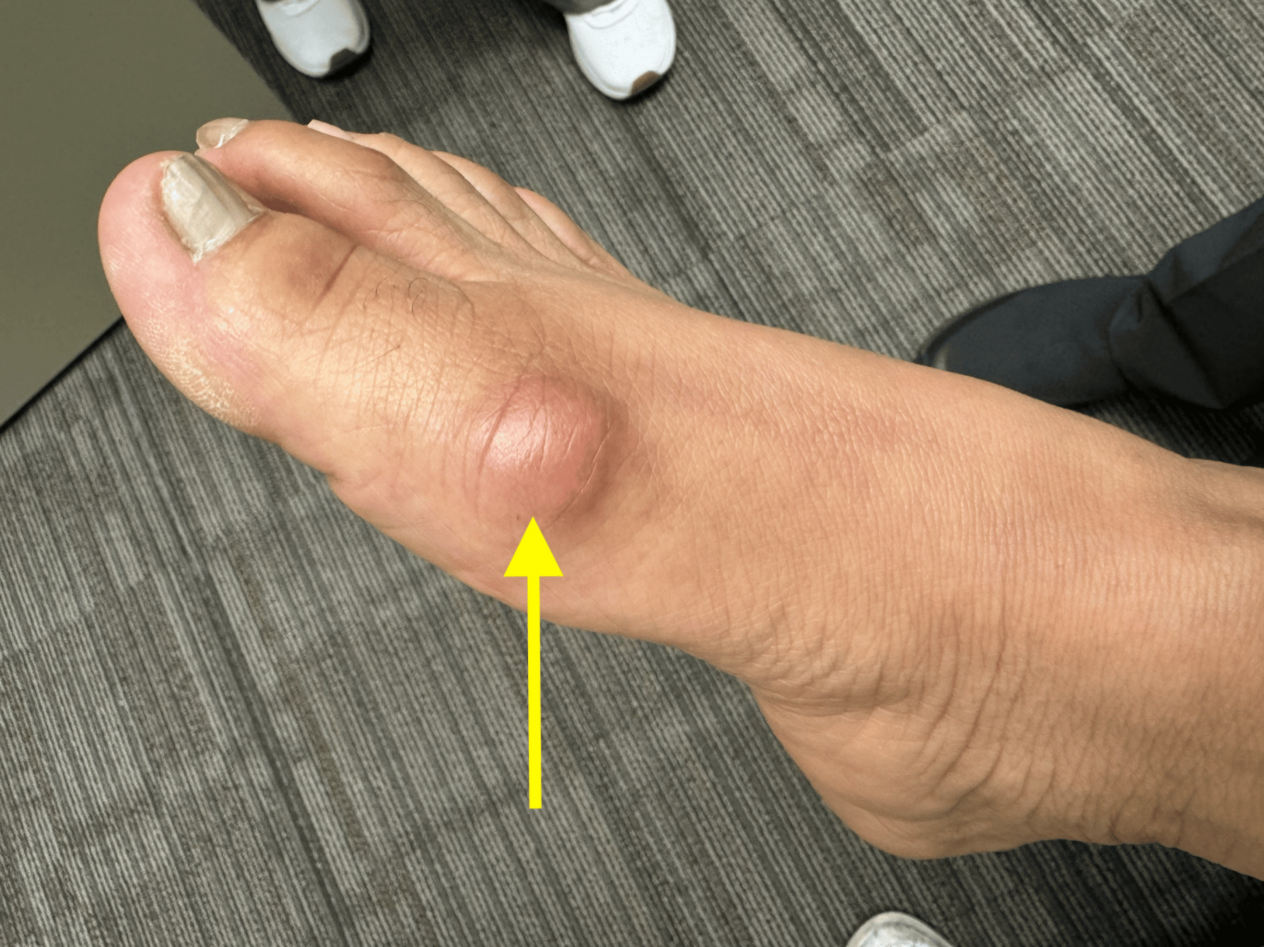
Subcutaneous mass over right first metatarsal head (arrow)

The patient returned to the clinic nine months later with severe, intermittent right hallux pain. Examination revealed a mass over the right first metatarsal head without significant erythema or signs of infection (Figure 1). The remainder of her physical exam remained unchanged from previous visits. Laboratory evaluation of serum calcium, phosphorus, uric acid, and ESR was normal. Furthermore, there was no evidence of leukocytosis, and blood cultures did not isolate any pathogens. The differential diagnosis considered possible bacterial infection or inflammatory processes such as gout. Initial management with broad-spectrum antibiotics, colchicine, and anti-inflammatory medications was unsuccessful.

As her pain symptoms became persistent, the patient presented to the emergency department for evaluation. Radiography revealed a calcified lesion at the site of the palpated mass. Following referral to podiatry, the lesion was surgically excised. Histopathologic analysis of the surgical specimens identified proliferative synovial tissue, calcified fibrous tissue, and no evidence of acute or chronic inflammatory infiltrate. This finding is consistent with dystrophic calcification, the underlying pathologic process responsible for calcinosis in the setting of CREST syndrome [6]. In the broader context of her autoimmune disease, the presence of calcinosis confirms the complete expression of all five hallmark features. Due to institutional limitations within the community-based clinic where the patient was seen, original radiologic imaging and histopathologic photographs are not available for publication. However, formal reports confirming these findings were documented in the patient’s medical record.

## Discussion

While the diagnosis of CREST syndrome requires at least three of five diagnostic criteria, this case is significant for the complete disease manifestation as well as the rapid progression of symptoms. A majority of patients with this condition develop the signs and symptoms over many years, particularly Raynaud’s phenomenon, which can appear years before other symptoms [1,4]. In contrast, the female patient in the current report demonstrated calcinosis, Raynaud’s phenomenon, esophageal dysmotility, sclerodactyly, and telangiectasia within a one-year period. This uncommon disease trajectory underscores the importance of heightened clinical suspicion of autoimmune disorders, especially in patients with limited healthcare access.

There were multiple social factors that complicated this patient’s treatment course and clinical work-up. Because she spoke only Spanish, there was a significant communication barrier to overcome. While language interpretation services were utilized, miscommunication was likely. The patient also struggled with food insecurity and an intermittent lack of health insurance. These factors likely hindered her ability to maintain consistent follow-up, adhere to treatment, and initiate early physical therapy. Her recent history of endometrial cancer further complicated her care by diverting clinical attention away from autoimmune disease management. The compounding effects of her social determinants of health likely contributed to delayed assessment and eventual surgical excision of her lower extremity calcinosis. Now established at the community health clinic, the patient has greater access to medical care and social services.

Treatment of CREST syndrome focuses primarily on symptom management and is tailored to the patient’s specific disease manifestations. Calcinosis is typically managed with supportive care, including pain control, anti-inflammatory medications, and, in refractory cases, surgical excision [6]. First-line treatment for Raynaud’s phenomenon includes calcium channel blockers and other vasodilators [4]. Esophageal dysmotility, presenting in this patient as gastroesophageal reflux disease (GERD) symptoms, is commonly addressed with proton pump inhibitors (PPIs) [1]. For sclerodactyly, immunosuppressants such as methotrexate may help delay progressive fibrosis [7], while physical and occupational therapy play a crucial role in preserving hand function [1]. Telangiectasia is generally treated for cosmetic purposes, often with pulsed dye laser therapy [1]. A multidisciplinary approach is also essential for these patients, particularly for monitoring systemic involvement and long-term disease progression. Early and regular screening for pulmonary complications, such as interstitial lung disease and pulmonary hypertension, with HRCT is critical even in asymptomatic patients [1].

In this patient’s case, long-term success will depend on continued engagement with her multidisciplinary care team. Support from primary care, rheumatology, pulmonology, and social services will be essential in addressing her condition and barriers to care.

## Conclusions

This case demonstrates a rare and complete presentation of CREST syndrome, identified by all five diagnostic criteria (calcinosis, Raynaud’s phenomenon, esophageal dysmotility, sclerodactyly, telangiectasia) as well as positive serology (ANA, anti-centromere). This case was further atypical due to her rapid disease progression within approximately 12 months, while symptoms generally progress over multiple years. Given this unusual course, clinicians should consider early serologic testing for patients presenting with Raynaud’s phenomenon and overlapping systemic disease manifestations. 

Equally important are the social factors that significantly impacted this patient’s healthcare, both in terms of access and treatment compliance. She faced multiple barriers, including language differences, a lack of consistent health insurance, and food insecurity. These socioeconomic challenges are not uncommon in chronic disease patients, necessitating recognition to improve disease morbidity and overall burden. 

In summary, this case presentation showcases a rare, full expression of CREST syndrome with rapid disease progression. It reinforces the importance of early symptom identification, serologic testing, pulmonary screening, and a multidisciplinary approach to improve outcomes in underserved populations. 
